# Supplementary material for: Identification of photoperiod-induced specific miRNAs in the adrenal glands of Sunite sheep (Ovis aries)
Source: Front Vet Sci. 2022 Jul 22;9:888207. doi: 10.3389/fvets.2022.888207 (PMC9354845; doi:10.3389/fvets.2022.888207)
Supplement: Supplementary file 1 [file Data_Sheet_1.ZIP › Supplementary file/miRDeep2 file.zip!/miRDeep2 file/Novel_Structure/SPLP42A1/Novel_272.pdf]

Provisional ID	:	NC_019470.2_19253
Score total	:	1.8
Score for star read(s)	:	-1.3
Score for read counts	:	0
Score for mfe	:	1.5
Score for randfold	:	1.6
Score for cons. seed	:	
Total read count	:	33
Mature read count	:	32
Loop read count	:	0
Star read count	:	1

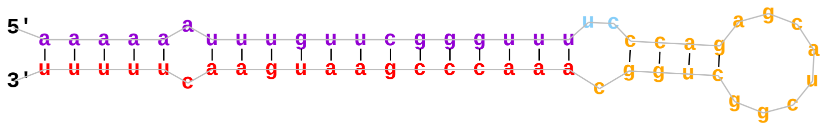

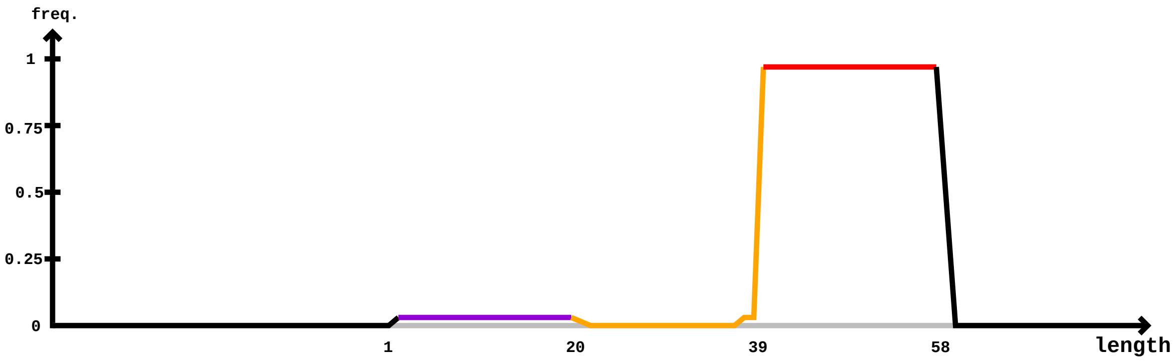


```
                                       Star                           Mature
5'- guguugaucuaacacacuauaucucagguuggucaaaaaauuuguucggguuuucccagagcaucggcuggcaaacccgaaugaacuuuuuggucaacucacuacucuggca   -3'    obs
    guguugaucuaacacacuauaucucagguuggucaaaaaauuuguucggguuuucccagagcaucggcuggcaaacccgaaugaacuuuuuggucaacucacuacucuggca          exp
    ((((((...)))))))...........(((((((((((((.(((((((((((((..((((........)))).))))))))))))).)))))))))))))).............   reads   mm   sample
    ..................................aaaaaGuuuguucggguuu...........................................................   1       1    seq
    ......................................................................gcaaacccgaaugaacuuuuug....................   1       0    seq
    ........................................................................aaacccgaaugaacuuuuu.....................   26      0    seq
    ........................................................................aaacccgaaugaacuuuuC.....................   1       1    seq
    ........................................................................aaacccgaaugaacuuuuCg....................   2       1    seq
    ........................................................................aaaccGgaaugaacuuuuug....................   1       1    seq
    ........................................................................aaacccgaaugaaUuuuuug....................   1       1    seq
```
